# *Ficus insipida* subsp. *insipida* (Moraceae) reveals the role of ecology in the phylogeography of widespread Neotropical rain forest tree species

**DOI:** 10.1111/jbi.12326

**Published:** 2014-05-14

**Authors:** Eurídice N Honorio Coronado, Kyle G Dexter, Monica F Poelchau, Peter M Hollingsworth, Oliver L Phillips, R Toby Pennington, Mark Carine

**Affiliations:** 1 School of Geography, University of LeedsLeeds, LS2 9JT, UK; 2 Instituto de Investigaciones de la Amazonia PeruanaIquitos, Peru; 3 School of GeoSciences, University of EdinburghEdinburgh, EH9 3JN, UK; 4 Royal Botanic Garden EdinburghEdinburgh, EH3 5LR, UK; 5 Department of Biology, Georgetown UniversityWashington, DC, 20057, USA

**Keywords:** Amazonia, Andes, genetic diversity, lineage divergence, Mesoamerica, phylogeographical structure, pioneer species, pollen dispersal, seasonally dry vegetation, seed dispersal

## Abstract

**Aim:**

To examine the phylogeography of *Ficus insipida* subsp. *insipida* in order to investigate patterns of spatial genetic structure across the Neotropics and within Amazonia.

**Location:**

Neotropics.

**Methods:**

Plastid DNA (*trn*H–*psb*A; 410 individuals from 54 populations) and nuclear ribosomal internal transcribed spacer (ITS; 85 individuals from 27 populations) sequences were sampled from Mexico to Bolivia, representing the full extent of the taxon's distribution. Divergence of plastid lineages was dated using a Bayesian coalescent approach. Genetic diversity was assessed with indices of haplotype and nucleotide diversities, and genetic structure was examined using spatial analysis of molecular variance (SAMOVA) and haplotype networks. Population expansion within Amazonia was tested using neutrality and mismatch distribution tests.

**Results:**

*trn*H–*psb*A sequences yielded 19 haplotypes restricted to either Mesoamerica or Amazonia; six haplotypes were found among ITS sequences. Diversification of the plastid DNA haplotypes began *c*. 14.6 Ma. Haplotype diversity for *trn*H–*psb*A was higher in Amazonia. Seven genetically differentiated SAMOVA groups were described for *trn*H–*psb*A, of which two were also supported by the presence of unique ITS sequences. Population expansion was suggested for both markers for the SAMOVA group that contains most Amazonian populations.

**Main conclusions:**

Our results show marked population genetic structure in *F. insipida* between Mesoamerica and Amazonia, implying that the Andes and seasonally dry areas of northern South America are eco-climatic barriers to its migration. This pattern is shared with other widespread pioneer species affiliated to wet habitats, indicating that the ecological characteristics of species may impact upon large-scale phylogeography. *Ficus insipida* also shows genetic structure in north-western Amazonia potentially related to pre-Pleistocene historical events. In contrast, evident population expansion elsewhere in Amazonia, in particular the presence of genetically uniform populations across the south-west, indicate recent colonization. Our findings are consistent with palaeoecological data that suggest recent post-glacial expansion of Amazonian forests in the south.

## Introduction

Phylogeographical studies can give insights into past changes in species distributions that can be related to environmental change and the history of landscapes (Avise, 2000). Phylogeography therefore has much to offer in understanding past vegetation dynamics in areas where macro- and microfossil records are rare. For this reason, there have been an increasing number of phylogeographical studies of trees in the Neotropics (reviewed by Cavers & Dick, 2013). While these studies have given insights into large-scale geographical relationships of populations, their results have not always been consistent. Given the massive geographical scale of Neotropical forests, especially Amazonia, which contain the most species-rich forests in the world (Gentry, 1988), much work remains to be done.

At a broad-scale, low genetic structure has been inferred from nuclear markers of pioneer species across the Neotropics indicating high gene flow for pollen and/or seeds and/or recent colonization over large areas (Dick *et al*., 2007; Turchetto-Zolet *et al*., 2012; Rymer *et al*., 2013; Scotti-Saintagne *et al*., 2013a). However, the genetic structure inferred from the plastid genome is not consistent across study species. Given that the plastid genome is inherited maternally in most angiosperms, this pattern implies that the seed dispersal history of these species varies.

Ecological characteristics of species may contribute to this variation in phylogeographical patterns. For example, studies of *Ceiba pentandra* (Dick *et al*., 2007), *Cordia alliodora* (Rymer *et al*., 2013) and *Jacaranda copaia* (Scotti-Saintagne *et al*., 2013a) indicate that these long-lived pioneer trees with wind-dispersed seeds and tolerance to drought can overcome two potential barriers between Amazonia and Mesoamerica: the Andean Cordillera and seasonally dry areas of northern South America. These studies show weak phylogeographical structure for plastid markers across the Neotropics, suggesting recent seed dispersal across these barriers. In contrast, other pioneer species with wind-dispersed seeds, notably *Schizolobium parahyba* (Turchetto-Zolet *et al*., 2012) and *Ochroma pyramidale* (Dick *et al*., 2013), both intolerant to drought, show genetic evidence for restricted seed dispersal between populations located at either side of these barriers. These contrasting patterns may imply that whether or not Neotropical rain forest tree species are drought-tolerant may have a strong impact on their phylogeography.

Within Amazonia, prior phylogeographical studies of trees have used intense sampling in a restricted geographical location (e.g. a 250-km transect; Dexter *et al*., 2012), in a region representing just part of a species' range (1500- to 2500-km transect; Lemes *et al*., 2010; Turchetto-Zolet *et al*., 2012), or sparse population sampling across a wide geographical range (Dick *et al*., 2007; Dick & Heuertz, 2008). Only a few studies have moderate, range-wide sample densities in Amazonia (e.g. Rymer *et al*., 2013; Scotti-Saintagne *et al*., 2013a,b). Collectively, this work has demonstrated contrasting phylogeographical patterns across the Amazon Basin inferred from the plastid genome. For example, plastid genetic differentiation measured in multiple species of *Inga* (Fabaceae) in south-eastern Peru suggests a zone of secondary contact between two historically isolated populations (Dexter *et al*., 2012). High genetic differentiation was also reported among populations of *Swietenia macrophylla* in the Brazilian Amazon using chloroplast microsatellite data (Lemes *et al*., 2010). In contrast, *Ceiba pentandra* (Dick *et al*., 2007) and *Symphonia globulifera* (Dick & Heuertz, 2008) have low genetic structure for plastid DNA markers across Amazonia.

Strong genetic structure in areas of Amazonia that are currently covered in continuous rain forest and without evident barriers to migration may reflect the effects of historical events in generating isolation among populations (Dexter *et al*., 2012). These historical events could include large fluvial rearrangements during the late Miocene (Hoorn *et al*., 1995), high frequency of fluvial dynamics of lateral erosion and deposition during the Pliocene–Pleistocene (Salo *et al*., 1986), or Quaternary climatic fluctuations (Haffer, 1969). Low genetic structure across the vast Amazon Basin in other species is consistent with recent population expansion (Dick & Heuertz, 2008). Nevertheless, comprehensive evaluation of population genetic structure across the range of widely distributed Amazon species is a demanding task.

Here, we examine the phylogeography of *Ficus insipida* Willd. subsp. *insipida* (Moraceae) at a broad-scale across the Neotropics and at a regional-scale within Amazonia. This subspecies is a key element of early successional Neotropical rain forest communities. It is a good exemplar taxon for widespread Neotropical rain forest trees because it is distributed across the Andes and into Mesoamerica, is a long-lived pioneer tree confined to humid environments, and, like the majority of rain forest trees, has animal-dispersed seeds. We used an intensive range-wide geographical sampling scheme and worked with both plastid and nuclear DNA markers. Our specific objectives were: (1) to estimate divergence time among lineages; (2) to compare genetic diversity within and among populations and between Mesoamerica and Amazonia; (3) to test population genetic structure and the spatial location of genetic breaks; (4) to establish where populations of *F. insipida* subsp. *insipida* show genetic uniformity or distinctiveness in Amazonia; and (5) to establish if the regional patterns we find might reflect Quaternary climate changes or the legacies of more ancient geological events.

## Materials and methods

### The study species and population sampling

The pantropical genus *Ficus* L. comprises *c*. 750 species (Berg, 2001). *Ficus insipida* is the most morphologically distinct and widespread Neotropical species of the section *Pharmacosycea*, a group of *c*. 20 tree species (Berg, 2001). In this study, we focus on *F. insipida* subsp. *insipida*, which is distributed from Mexico through the Andean region to the lowland rain forest of western Amazonia. This taxon is easily identified by its oblong to elliptic, bright and shiny leaves with yellow secondary veins and 5–12.5 cm long terminal stipules (Berg *et al*., 1984). *Ficus insipida* subsp. *scabra* C.C. Berg from eastern Brazil, the Guianas and north-eastern Venezuela, and *Ficus adhatodifolia* Schott from southern Bolivia, southern Brazil (including the Atlantic rain forest) and Paraguay have similar leaf morphology to *F. insipida* subsp. *insipida* but have smaller stipules (1–4 cm long), different coloration in the mature fruits, and non-overlapping geographical ranges (Berg *et al*., 1984). We did not find these taxa in our sampling areas, and consider that problems of misidentification in our sampling are minimal.

Our study is based principally on leaf samples collected from the field for 410 individual trees of *F. insipida* subsp. *insipida*. A total of 54 populations from Mexico, Belize, El Salvador, Nicaragua, Costa Rica, Panama, Ecuador, Peru and Bolivia were visited, covering the breadth of the taxon's distribution across Mesoamerica (*n *=* *31 sites) and Amazonia (*n *=* *23 sites). At least eight individuals were collected at each site, although fewer samples were sourced from several sites where the species was rare. In some cases our sampling was supplemented using herbarium specimens. Leaf samples were dried and stored in silica gel and the locations of individuals were recorded using a handheld GPS. For Amazonian populations, at least one herbarium voucher was collected from each population (see Appendix S1 in Supporting Information).

### DNA extraction, sequencing and editing

Total genomic DNA was extracted using the CTAB method (Doyle & Doyle, 1987). Seven plastid markers were tested for amplification and sequence variation: *rpl*32*–trn*L*, trn*Q–5′-*rps*16, 3′*trn*V–*nd*HC, *atp*I–*atp*H, *trn*D–*trn*T, *trn*H–*psb*A and *trn*L–*trn*F (Shaw *et al*., 2007). The non-coding marker *trn*H*–psb*A was chosen for the full-scale study because of its high amplification success and variability within and among populations of *F. insipida* subsp. *insipida*. In addition, the internal transcribed spacer region (ITS) of the nuclear ribosomal DNA was amplified and sequenced for 77 samples from Amazonia using the ITS1 and ITS4 primers (White *et al*., 1990). Eight additional ITS sequences from other regions (one from Mexico, one from Costa Rica, five from Panama and one from Brazil) were contributed by collaborators (see Acknowledgements). Reaction conditions varied slightly between Mesoamerican and Amazonian samples, because amplifications were performed in different laboratories. Mesoamerican PCR reactions were performed in 10 μL volume, and contained 1 μL of template DNA, 1× Sigma PCR buffer, 3.0 mm MgCl_2_, 200 μm of each dNTP, 0.4 μL 10 mg/mL bovine serum albumin (BSA), 0.2 μm of each primer and 1 U JumpStartTM Taq polymerase (Sigma Aldrich, St. Louis, MO, USA) (see Poelchau & Hamrick, 2013). PCR for the Amazonian samples was performed in 20 μL solutions containing 2 μL of template DNA, 2 μL of PCR Buffer 10×, 2 μL of 10 mm total dNTP, 1 μL of 50 mm MgCl_2_, 1 μL of each primer, 4 μL of combinatorial enhancer solution (CES), 0.2 μL of Taq polymerase (Bioline, UK) and 6.8 μL of distilled H_2_O. However, identical cycling programs were used for samples of both geographical regions. The thermal cycle for *trn*H–*psb*A (and ITS) was 94 °C for 5 min (3 min), followed by 35 (30) cycles at 94 °C for 30 s (1 min), 55 °C (56 °C) for 30 s (1 min) and 72 °C for 1 min (90 s), and a final extension at 72 °C for 10 min (5 min). Mesoamerican PCR products were sequenced as in Poelchau & Hamrick (2013); Amazonian PCR products were visualized via 1% agarose gel electrophoresis, and products were purified using ExoSAP-IT (Affymetrix UK Ltdn, High Wycombe, UK). Cycle sequencing was conducted in 10 μL solutions containing 3 μL PCR product, 0.5 μL of BigDye (Applied Biosystems, Paisley, UK), 2 μL sequencing reaction buffer 5×, 0.32 μL of primer and 4.18 μL of distilled H_2_O.

All forward and reverse strands were edited in Sequencher 5.0 (Gene Codes Corporation, Ann Arbor, MI, USA) and nucleotide substitutions, indels (i.e. insertions or deletions) and inversions were visually checked against the original electropherograms. The alignment was created manually in Mesquite 2.74 (Maddison & Maddison, 2001). Mononucleotide repeat polymorphisms were excluded from all subsequent analyses.

### Statistical analyses

#### Divergence of plastid lineages

Bayesian inference was used to estimate divergence time among plastid DNA haplotypes. The ingroup comprised all plastid haplotypes of *F. insipida* subsp. *insipida*. Sequences downloaded from GenBank representing nine *Ficus* species of sections *Pharmacosycea* (GQ982221, GQ982222, GQ982225GQ982227), *Americana* (GQ982218, GQ982219 and GQ982224), *Sycomorus* (EU213825) and *Galoglychia* (EU213821) and *Poulsenia armata* (tribe Castilleae) were used as multiple outgroups. Inversions and indels were excluded from the analysis.

Phylogenetic reconstruction of the plastid sequences was performed in beast 1.6.2 (Drummond & Rambaut, 2007) using the uncorrelated lognormal relaxed molecular clock and the HKY nucleotide substitution model that was the closest model suggested by jModelTest 0.0.1 (Posada, 2008). The tree prior model was set using a coalescent approach assuming constant population size. A normal distribution was used for the prior on tree root age with a mean value of 73.9 Ma and a standard deviation including the age estimates for the divergence between *Ficus* and *Poulsenia* (49.6–88.2 Ma; Zerega *et al*., 2005). Three replicate runs were performed using a chain of 100,000,000 states sampling every 10,000 generations. All trees were combined after the exclusion of the first 1000 trees of each run as burn-in to avoid including trees sampled before convergence of the Markov chains. The posterior probabilities and ages of nodes were thus derived from a posterior distribution of 27,000 total trees.

#### Genetic diversity and population structure

**Figure 1:**
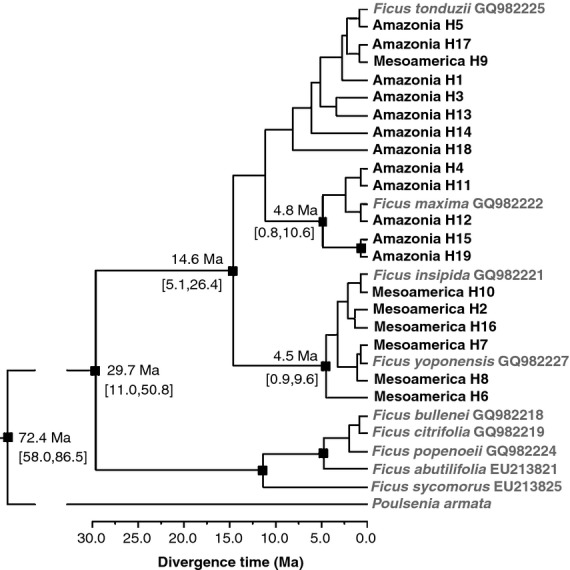
Lineage divergence dating for all plastid DNA haplotypes (H1–H19) of *Ficus insipida* subsp. *insipida* occurring in Mesoamerica and Amazonia. Mean divergence dates are given with 95% highest posterior density values in brackets. Nodes with posterior probabilities above 0.95 are indicated with a black square. Sequences downloaded from GenBank are indicated in grey.

The phylogenetic analysis suggested that three outgroup taxa, *Ficus maxima, Ficus tonduzii* and *Ficus yoponensis*, are nested within *F. insipida* subsp. *insipida* (Fig.1). These species are highly distinctive morphologically from *F. insipida* subsp. *insipida*, which we consider to be a reproductively isolated biological species, and we therefore conducted all population genetic analyses on populations of this taxon alone. The pattern of phylogenetic nesting may simply be due to the origin of one or more of these species from populations of *F. insipida* subsp. *insipida*, although the possibility that *F. maxima*, *F. tonduzii* and *F. yoponensis* are more distantly related and sharing haplotypes by occasional hybridization requires consideration (see Results and Discussion).

The genealogical relationships of haplotypes were estimated independently for *trn*H*–psb*A and ITS sequences using statistical parsimony in tcs 1.21 (Clement *et al*., 2000), with a 95% parsimony connection limit. Individual indels and inversions were treated as single mutation events.

Because of highly uneven sampling of ITS sequences between Mesoamerica and Amazonia, all subsequent analyses were performed only for *trn*H*–psb*A sequences, while the ITS data were used for comparison of general patterns. Haplotype and nucleotide diversities were calculated for each population in Arlequin 3.5 (Excoffier & Lischer, 2010). We compared genetic diversity between Mesoamerica and Amazonia using a rarefaction procedure set to 100 runs that standardized each region to the same number of sequences (*n *=* *182 individuals) using the packages ape (Paradis *et al*., 2004) and pegas (Paradis, 2010) in R Statistical Software, version 2.15.2. A pattern of isolation by distance for the plastid marker (Wright, 1943), which can indicate restricted seed dispersal, was assessed using a Mantel test in R Statistical Software. Specifically, we compared Nei's pairwise genetic distance among populations and the logarithm of the Euclidean geographical distances. The significance of the relationship was tested with 10,000 permutations.

Genetic differentiation among populations was estimated by computing a distance matrix based on the number of mutational steps between haplotypes (*N*_ST_) and by using haplotype frequencies (*G*_ST_). The presence of a phylogenetic component to the phylogeographical structure was assessed by testing whether *N*_ST_ is significantly higher than *G*_ST_ based on 10,000 permutations in PermutCpSSR 2.0 (Pons & Petit, 1996). A spatial analysis of molecular variance (SAMOVA) was performed to determine the position of genetic breaks among populations using samova 1.0 (Dupanloup *et al*., 2002). Several runs were performed using increasing numbers of groups (*K *=* *1–20) and 100 annealing simulations for each *K*. In each run, populations were clustered into genetically and geographically homogenous groups (Dupanloup *et al*., 2002). The number of groups was chosen so as to maximize genetic differentiation among the groups (Φ_CT_). Genetic structure among groups of populations defined by geographical region and by SAMOVA was further examined by analysis of molecular variance computing a distance matrix in Arlequin 3.5. Significance of genetic structure indices was tested using a nonparametric randomization procedure.

#### Demographic history

Within Amazonia, the population expansion hypothesis was tested for plastid and nuclear DNA sequences in Arlequin 3.5. We used Fu's *F*_*S*_ neutrality test, based on the number of pairwise differences, because this statistic shows large negative values under population expansion (Fu, 1997). In addition, we used a mismatch distribution test to assess whether the observed distribution of pairwise differences matches the expectations under a model of population expansion (Schneider & Excoffier, 1999). We also estimated the parameter tau of demographic expansion (τ) using a generalized nonlinear least squares approach. If a demographic expansion model is not rejected (*P *>* *0.05), time since the expansion (*t*) can be calculated as τ =2μ*t*, where μ is the mutation rate for the total length of each DNA marker. A mean mutation rate of 1.64 × 10^−9^ substitutions site^−1^ year^−1^ (s s^−1 ^yr^−1^) for ITS was taken from Dick *et al*. (2013), while a mean value for the plastid marker was taken from the divergence time analysis. These tests were performed for each SAMOVA group in Amazonia.

## Results

### Non-monophyly of *F. insipida* subsp. *insipida*

The phylogenetic analysis showed that *F. insipida* subsp. *insipida* is not monophyletic; other *Ficus* species of section *Pharmacosycea*, *F. maxima, F. tonduzii* and *F. yoponensis,* are nested within it (Fig.1). These three taxa are readily morphologically distinguished from *F. insipida* subsp. *insipida*, and have similarly wide ranges spanning both Central and South America (see http://www.tropicos.org/). The single accessions representing each of these three species come from Barro Colorado Island, Panama (Mesoamerica), but *F. tonduzii* and *F. maxima* have sequences related to Amazonian haplotypes of *F. insipida* and therefore do not cluster geographically with Mesoamerican *F. insipida* haplotypes. Finally, a GenBank accession of *F. insipida* subsp. *insipida* (GQ982221) also collected in Barro Colorado Island is nested within Mesoamerican *F. insipida* haplotypes (Fig.1).

### Divergence time

Diversification of plastid DNA haplotypes in *F. insipida* subsp. *insipida* appears to have begun in the Miocene with a split into Mesoamerican and Amazonian lineages estimated at 14.6 Ma (95% highest posterior density, HPD: 5.1–26.4 Ma; Fig.1; see phylogram in Appendix S2a). Pliocene ages were estimated for the main diversification of Mesoamerican haplotypes (4.5 Ma; 95% HPD: 0.9–9.6 Ma; Fig.1) and for the haplotypes H4, H11, H12, H15 and H19 of northern Amazonia (4.8 Ma; 95% HPD: 0.8–10.6 Ma). A second Mesoamerican lineage corresponding to haplotype H9 was nested within the most diverse clade of Amazonian lineages (Fig.1). The mean substitution rate obtained using the relaxed molecular clock was 4.40 × 10^−10^ s s^−1^ yr^−1^ (95% HPD: 2.41 × 10^−10^ to 6.62 × 10^−10^ s s^−1^ yr^−1^).

### Plastid DNA

**Figure 2:**
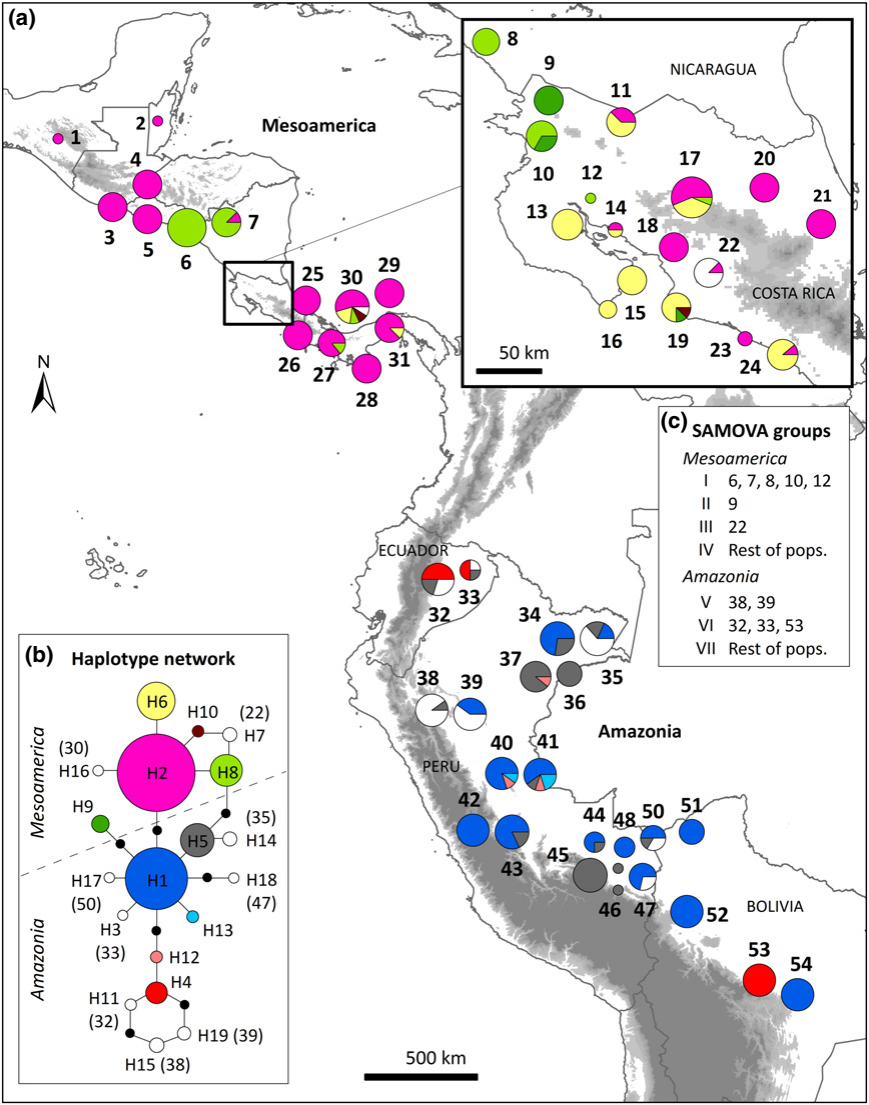
(a) Haplotype distribution, (b) haplotype network, and (c) SAMOVA groups of *trn*H*–psb*A sequences for *Ficus insipida* subsp. *insipida* populations sampled from 54 sites in Mesoamerica and Amazonia. Haplotype distributions at the border between Costa Rica and Nicaragua are shown separately. Pie charts are labelled with population numbers as shown in Table1. Colours represent the haplotypes (H1–H19). In the haplotype network (b), haplotypes unique to a single population are shown in white with population number given in brackets. Circle size is proportional to sample size for each population (*n *=* *1–16 individuals) and for each haplotype (*n *=* *1–119 individuals). Missing haplotypes in the network are shown as black dots, and a dashed line separates haplotypes of each region. The Andean Cordillera and other mountains are shown in shaded grey.

For the plastid *trn*H*–psb*A marker, a total of 410 individuals collected in 54 populations were successfully sequenced (Table1): 228 samples from Mesoamerica (*n *=* *31 sites) and 182 from Amazonia (*n *=* *23 sites). After the exclusion of two variable mononucleotide repeats, 340 bp of aligned sequences remained. A total of 19 polymorphic sites were detected including 14 substitutions, four indels and one inversion (see Appendix S3); these mutations defined a total of 19 haplotypes (Fig.2a). The haplotypes were geographically restricted; seven were confined to Mesoamerica and twelve to Amazonia (Fig.2b). The analysis at the population level indicates values of haplotype diversity above 0.70 in populations 30, 33 and 50, and of nucleotide diversity above 0.70% in populations 10, 33 and 39 (Table1). In addition, rarefied haplotype diversity was higher in Amazonia (95% confidence interval, CI: 0.694–0.703) than Mesoamerica (95% CI: 0.646–0.654), while rarefied nucleotide diversity was higher, but not significantly so in Amazonia (95% CI: Amazonia 0.328%–0.404%; Mesoamerica 0.281%–0.348%).

**Table 1:** Haplotype diversity and nucleotide diversity (mean ± SD for both indices) for the plastid *trn*H–*psb*A marker in 54 *Ficus insipida* subsp. *insipida* populations in Mesoamerica and Amazonia. The metrics were not applicable for populations with less than three individuals sampled. The number of sequences is provided for each population. Regional genetic diversity was estimated using rarefaction procedure.

No.	Code	Country	Ind.	Hapl. div.	Nucl. div. (%)
1	MEX	Mexico	1	n.a.	n.a.
2	BEL	Belize	1	n.a.	n.a.
3	ElI	El Salvador	8	0	0
4	Dei	El Salvador	8	0	0
5	Nan	El Salvador	8	0	0
6	VoC	Nicaragua	14	0	0
7	Mir	Nicaragua	8	0.25 ± 0.18	0.07 ± 0.11
8	ElO	Nicaragua	7	0	0
9	HLI	Costa Rica	8	0	0
10	RiT	Costa Rica	9	0.50 ± 0.13	0.74 ± 0.50
11	CaN	Costa Rica	8	0.54 ± 0.12	0.16 ± 0.17
12	RiB	Costa Rica	1	n.a.	n.a.
13	RiN	Costa Rica	9	0	0
14	LaE	Costa Rica	2	n.a.	n.a.
15	Cur	Costa Rica	8	0	0
16	CaB	Costa Rica	3	0	0
17	RSC	Costa Rica	16	0.58 ± 0.08	0.19 ± 0.17
18	Esp	Costa Rica	8	0	0
19	Jac	Costa Rica	8	0.46 ± 0.20	0.50 ± 0.37
20	LaS	Costa Rica	8	0	0
21	EaU	Costa Rica	8	0	0
22	Car	Costa Rica	8	0.25 ± 0.18	0.15 ± 0.16
23	MaA	Costa Rica	2	n.a.	n.a.
24	HaB	Costa Rica	9	0.22 ± 0.17	0.07 ± 0.10
25	Cah	Costa Rica	8	0	0
26	PiB	Costa Rica	8	0	0
27	CeB	Panama	7	0.29 ± 0.20	0.09 ± 0.12
28	LaT	Panama	8	0	0
29	FtS	Panama	8	0	0
30	PLR	Panama	11	0.71 ± 0.14	0.26 ± 0.22
31	PNM	Panama	8	0.25 ± 0.18	0.07 ± 0.11
32	JaS	Ecuador	10	0.69 ± 0.10	0.48 ± 0.35
33	Bog	Ecuador	4	0.83 ± 0.22	0.89 ± 0.69
34	Yan	Peru	11	0.44 ± 0.13	0.13 ± 0.14
35	Mad	Peru	11	0.58 ± 0.14	0.25 ± 0.22
36	SaJ	Peru	6	0	0
37	JeH	Peru	9	0.22 ± 0.17	0.20 ± 0.19
38	Mar	Peru	10	0.20 ± 0.15	0.30 ± 0.25
39	Ura	Peru	10	0.53 ± 0.09	0.79 ± 0.52
40	vHu	Peru	10	0.38 ± 0.18	0.18 ± 0.17
41	Mac	Peru	10	0.64 ± 0.15	0.28 ± 0.24
42	LaG	Peru	10	0	0
43	SaT	Peru	11	0.33 ± 0.15	0.10 ± 0.12
44	CoC	Peru	4	0.50 ± 0.27	0.15 ± 0.18
45	Qon	Peru	11	0	0
46	SaG	Peru	1	n.a.	n.a.
47	Tam	Peru	7	0.48 ± 0.17	0.28 ± 0.25
48	LoA	Peru	4	0	0
49	LaP	Peru	1	n.a.	n.a.
50	Tah	Bolivia	6	0.73 ± 0.16	0.26 ± 0.24
51	Aba	Bolivia	6	0	0
52	Mai	Bolivia	10	0	0
53	Sac	Bolivia	10	0	0
54	LaEn	Bolivia	10	0	0
MESOAMERICA			228	0.65 ± 0.03	0.31 ± 0.23
AMAZONIA			182	0.70 ± 0.03	0.37 ± 0.26

n.a., not applicable.

### Population structure

The Mantel test between genetic and geographical distance showed significant isolation by distance for the plastid marker (*r *=* *0.47, *P *<* *0.001), with higher mean genetic distance between populations in different regions (0.18) than within each region (Amazonia = 0.06 and Mesoamerica = 0.05). Genetic differentiation among populations was significantly higher when computed using a distance matrix (*N*_ST_ = 0.81) than when using haplotype frequencies (*G*_ST_ = 0.72, *P *<* *0.05), indicating some significant phylogeographical structuring. The SAMOVA analysis showed increasing values of differentiation among groups up to a *K* value of 7 (Φ_CT_ = 0.8). Four groups were defined in Mesoamerica, primarily segregating populations containing haplotypes H8 (group I), H9 (group II) and H7 (group III) from the rest of the Mesoamerican populations (group IV; Fig.2c). Three groups were found in Amazonia: group V containing two populations from north-eastern Peru and mainly haplotypes H15 and H19; group VI containing two populations from Ecuador and one from central Bolivia with mainly haplotype H4; and group VII containing the remaining Amazonian populations (Fig.2c). SAMOVA groups IV and VII contain most of the populations and are dominated by a few widespread haplotypes: H2 in Mesoamerica and H1 and H5 in Amazonia. Mesoamerican haplotype H9 is more related to Amazonian haplotypes, while haplotype H4 shows a disjunct distribution, occurring in Ecuador and in one population in Bolivia (Fig.2a). Grouping of populations by SAMOVA groups explained more of the genetic variation (75.1%) than grouping by the two overall geographical regions (56.2%; Table2).

**Table 2:** Analysis of molecular variance (AMOVA) based on pairwise differences of the plastid *trn*H*–psb*A marker for *Ficus insipida* subsp. *insipida*. The analysis was run independently using populations grouped by geographical regions (Mesoamerica and Amazonia) and by SAMOVA groups.

Group level	Source of variation	d.f.	Sum of squares	Variance components	Percentage of variation	Fixation indices (*P *<* *0.001)
Geographical regions	Among groups	1	203	0.98	56.21	Φ_CT_* *=* *0.56
	Among pops within groups	52	218	0.53	30.18	Φ_SC_* *=* *0.69
	Within populations	356	84	0.24	13.61	Φ_ST_* *=* *0.86
SAMOVA groups	Among groups	6	353	1.21	75.14	Φ_CT_* *=* *0.75
	Among pops within groups	47	68	0.16	10.13	Φ_SC_* *=* *0.41
	Within populations	356	84	0.24	14.73	Φ_ST_* *=* *0.85

Φ_CT_, genetic differentiation among groups.

Φ_SC_, genetic differentiation among populations within groups.

Φ_ST_, genetic differentiation among populations.

### Demographic analysis

Demographic analyses for the plastid and ribosomal nuclear DNA markers tentatively suggest range expansion in Amazonia mainly for SAMOVA group VII, which includes 18 of the 23 Amazonian populations. This group had negative values of Fu's *F*_*S*_ and low but non-significant *P-*values for both markers (Table3). In addition, the mismatch distribution for group VII was unimodal, with no significant deviation from the sudden demographic expansion model. Time since expansion was estimated as 2.69 Ma (95% CI: 1.85–4.12 Ma) for the *trn*H–*psb*A and as 1.44 Ma (95% CI: 0.23–1.68 Ma) for ITS (Table3).

**Table 3:** Demographic expansion tests performed for SAMOVA groups of *Ficus insipida* subsp. *insipida* in Amazonia. Time since the expansion was estimated using the mutation rate for the total length of each DNA marker (μ_*trn*H–*psb*A_* *=* *4.40 × 10^−10^ s s^−1 ^yr^−1^ × 340 bp, and μ_ITS_* *=* *1.64 × 10^−9^ s s^−1 ^yr^−1^ × 635 bp). Note that *P*-values for Fu's *F*_*S*_ are only considered significant at the 95% level if the *P*-value is lower than 0.02 (Excoffier & Lischer, 2010).

DNA marker	SAMOVA groups	Sample size	Neutrality test		Mismatch distribution		
			Fu's *F*_*S*_	*P*-value	τ	*P*-value	Time [95% CI]
*trn*H–*psb*A	V	20	2.51	0.89	6.99	0.16	
	VI	24	0.92	0.71	0.00	0.00	
	VII	138	−1.90	0.21	0.81	0.29	2.69 Ma [1.85–4.12]
ITS	V	9	0.00	n.a.	0.00	0.00	
	VI	10	0.59	0.04	3.00	0.06	
	VII	58	−2.21	0.03	3.00	0.14	1.44 Ma [0.23–1.68]

τ, parameter tau of demographic expansion; n.a., not applicable.

### Nuclear ribosomal DNA

**Figure 3:**
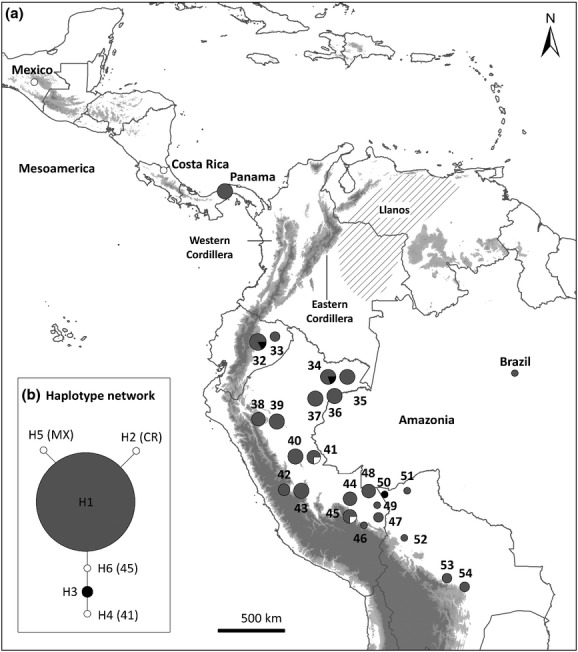
(a) Haplotype distribution and (b) haplotype network of ITS sequences for *Ficus insipida* subsp. *insipida* populations sampled from 27 sites in Mesoamerica and Amazonia. Pie charts are labelled with population numbers as shown in Table1. Colours represent the haplotypes (H1–H6). In the haplotype network (b), haplotypes unique to a single population are shown in white with population number given in brackets. Circle size is proportional to sample size for each population (*n *=* *1–6 individuals) and for each haplotype (*n *=* *1–76 individuals). The Andean Cordillera and other mountains are shown in shaded grey, and the dashed area represents the Llanos. Additional ITS sequences obtained from collaborators are indicated as Mexico (MX), Costa Rica (CR), Panama and Brazil (see Acknowledgements).

A total of 85 samples were sequenced for ITS, covering the entire study range, but focusing predominantly on Amazonia. Five polymorphic sites, three substitutions and two indels (Appendix S3) were found in the 635 bp of aligned sequences. These mutations defined six haplotypes of which haplotype H1 is widespread across Amazonia and extends to Panama in Central America. Haplotype H3 occurs in three localities in Amazonia and the remaining four haplotypes each occur in single populations in Amazonia and Mesoamerica (Fig.3).

## Discussion

### Non-monophyly of *Ficus insipida* subsp. *insipida*

Our phylogenetic analysis showed that three of the outgroup taxa, *F. maxima, F. tonduzii* and *F. yoponensis*, were nested within *F. insipida* subsp. *insipida* (Fig.1). The lack of monophyly is not unexpected given that *F. insipida* subsp. *insipida* and the related species are widespread, and may share ancestor–descendant relationships (cf. Gonzalez *et al*., 2009; Dexter *et al*., 2010). If species originate from within widespread species, it would take a long period of time for both to become reciprocally monophyletic in a gene tree (Avise, 2000) in the face of very large effective population size such as that of *F. insipida* subsp. *insipida*, which has a huge geographical range and is relatively common. We cannot definitively exclude the possibility that *F. maxima, F. tonduzii* and *F. yoponensis* are more distantly related to *F. insipida* subsp. *insipida* and that the pattern of shared haplotypes (Appendix S2b) is the result of hybridization with *F. insipida* subsp. *insipida*. However, we can at least conclude that hybridization is not frequent given that the species are morphologically distinct from other *Ficus* species, and intermediates have not been observed in the field (although this does not in itself exclude chloroplast capture). However, the accessions of *F. tonduzii* and *F. maxima* from Barro Colorado Island, Panama, share haplotypes with Amazonian accessions of *F. insipida* subsp. *insipida* (see Appendix S2b), and if the phylogeographical patterns we observe are due to hybridization we would expect to see geographical sharing of haplotypes. More fundamentally, the key patterns of the population genetic analyses of *F. insipida* subsp. *insipida* we observe and discuss below are robust regardless of concerns about species monophyly or the origin of haplotypes. These are a clear divide in chloroplast lineages between geographical areas, which can only be explained via lack of seed flow, and the presence of widespread haplotypes, which suggests recent population expansion.

### Genetic structure between Mesoamerica and Amazonia

We found clear population genetic structure in the plastid DNA data of *F. insipida* subsp. *insipida*, with differentiation between Mesoamerican and Amazonian haplotypes demonstrated by the haplotype network (Fig.2b) and AMOVA analysis (Table2). Moreover, there is a significant increase of genetic distance with geographical distance among populations, in particular for populations compared between geographical regions. Our phylogenetic analysis of the plastid DNA data is consistent with a split between Amazonian and Mesoamerican lineages of *F. insipida* subsp. *insipida* at 14.6 Ma (5.1–26.4 Ma) with subsequent dispersal from Amazonia to Mesoamerica only in the case of haplotype H9 (Fig.1). The early split of *F. insipida* subsp. *insipida* lineages implies that there has been limited genetic exchange via seeds and consequently very rare successful dispersal events between the regions. We discuss below at least two potential present-day barriers which can be invoked to explain limitation of seed dispersal between Mesoamerica and Amazonia.

The first and most obvious barrier is the Andes cordillera, which stretches along the entire western edge of South America with elevations (> 4000 m) greatly exceeding the current elevational limits of lowland rain forest trees. *Ficus insipida* subsp. *insipida* grows mainly in lowland rain forest and in pre-montane environments reaching elevations of 1500 m a.s.l. and with rare records at 1800–2000 m a.s.l. (see http://www.tropicos.org/). Therefore, the Andes are a significant potential dispersal barrier and must have been since 3 Ma when the most recent uplift of the Eastern Cordillera caused it to reach 2500 m a.s.l. (Gregory-Wodzicki, 2000). A second barrier for wet-adapted species is seasonally dry vegetation: extensive savannas in Colombia and Venezuela (the Llanos) and seasonally dry tropical forests along the Caribbean coast of northern South America (Pennington *et al*., 2006). *Ficus insipida* subsp. *insipida* cannot tolerate seasonal drought (Berg, 2001). Thus, the extensive seasonally dry areas in northern South America may prevent dispersal between Mesoamerica and Amazonia for rain forest trees such as *F. insipida* subsp. *insipida*.

Studies from other Neotropical tree species support the generalized interpretation that taxa which are unable to tolerate seasonally dry habitats have also been unable to use the northern seasonally dry tropical forest and savannas as a migration route. Limited seed flow via this route would explain their strong population differentiation between Mesoamerica and Amazonia. For example, *Schizolobium parahyba* (Turchetto-Zolet *et al*., 2012) and *Ochroma pyramidale* (Dick *et al*., 2013) are pioneer species restricted to wet environments, and show restricted seed dispersal between Mesoamerica and Amazonia (Table4). Moreover, shade-tolerant rain forest-confined species such as *Symphonia globulifera* (Dick & Heuertz, 2008), *Poulsenia armata* and *Garcinia madruno* (Dick *et al*., 2013) have distinct plastid DNA haplotypes in these regions. In contrast, weak population genetic structure with identical or closely related plastid DNA haplotypes spanning Mesoamerica and Amazonia have been reported for other pioneer trees such as *Ceiba pentandra* (Dick *et al*., 2007), *Cordia alliodora* (Rymer *et al*., 2013), *Jacaranda copaia* (Scotti-Saintagne *et al*., 2013a) and *Trema micrantha* (Dick *et al*., 2013). Although seed dispersal syndrome differs among these species, they have a broad ecological range and are tolerant to drought (Table4). In particular, *Ceiba pentandra*, *Cordia alliodora* and *Trema micrantha* are recorded in inventories of seasonally dry tropical forests on the Caribbean coast of Colombia (see herbarium collections at http://www.biovirtual.unal.edu.co/ICN/; Linares-Palomino *et al*., 2011). This apparent association between drought tolerance and levels of phylogeographical structure remains tentative until further detailed studies are available for comparison.

**Table 4:** Summary of phylogeographical studies of Neotropical pioneer tree species. Genetic markers used in each study including nuclear ribosomal DNA (nrDNA), chloroplast DNA (cpDNA), and nuclear and chloroplast simple sequence repeats (nuSSR and cpSSR, respectively) are indicated. Low genetic structure indicates similar nuclear or plastid DNA haplotypes between Mesoamerica and Amazonia while high structure indicates distinct haplotypes between these regions.

Species and family	Habitat	Genetic marker	Pollen dispersal	Seed dispersal	Genetic structure		Reference
					nrDNA	cpDNA	
*Ceiba pentandra* MALVACEAE	Wet to dry	nrDNA (ITS), cpDNA (*psb*B–*psb*F)	Vertebrates (bats) Insects (moths)	Wind and water	Low	Low	Dick *et al*. (2007)
*Cordia alliodora* BORAGINACEAE	Wet to dry	nrDNA (ITS), cpDNA (*trn*H–*psb*A), cpSSR	Insects (moths)	Wind	Low	Low	Rymer *et al*. (2013)
*Jacaranda copaia* BIGNONIACEAE	Wet to dry	cpDNA (*trn*H–*psb*A, *trn*C–*ycf6*), nuSSR, cpSSR	Insects (large bees)	Wind	N.A.	Low	Scotti-Saintagne *et al*. (2013a)
Ochroma pyramidale* MALVACEAE	Wet	nrDNA (ITS), cpDNA (*trn*H–*psb*A, *psb*B–*psb*F, *rbc*L)	Vertebrates (bats)	Wind	High	High	Dick *et al*. (2013)
*Schizolobium parahyba* FABACEAE	Wet	nrDNA (ITS), cpDNA (*trn*H–*psb*A, *trn*L–*trn*F, *mat*K)	Insects (bees)	Wind	Low	High	Turchetto-Zolet *et al*. (2012)
*Trema micrantha*[Table-fn tf4-1] ULMACEAE	Wet to dry	nrDNA (ITS), cpDNA (*trn*H–*psb*A, *psb*B–*psb*F, *rbc*L)	Small insects	Vertebrates (birds)	Low	Low	Dick *et al*. (2013)
*Ficus insipida* subsp. *insipida* MORACEAE	Wet	nrDNA (ITS), cpDNA (*trn*H–*psb*A)	Insects (wasps)	Vertebrates (fish, bats, others)	Low	High	This study

* Reduced sampling of species' distribution range; N.A., not available.

### Genetic structure within Amazonia

*Ficus insipida* subsp. *insipida* shows strong genetic differentiation in the northern part of the Amazon Basin where SAMOVA groups V and VI containing the populations from north-eastern Peru (codes 38 and 39), Ecuador (32 and 33) and Bolivia (53), are separated from SAMOVA group VII containing the remaining populations. The plastid haplotype found at Sacta (population 53 in Bolivia) may represent an extreme case of ancestral polymorphism, or alternatively, it may reflect a recent long-distance dispersal event from Ecuador, the only other area where we observed its presence. Despite the extensive sampling carried out in this study, it is also possible that this haplotype is present in the intervening area in unsampled trees. While the three SAMOVA groups have widespread haplotypes (e.g. H1 and H5), they additionally contain a haplotype set which is restricted to this area and belongs to a phylogenetically distinct clade that diversified approximately 4.8 Ma (e.g. H4 and H11 of Ecuador, H15 and H19 of north-eastern Peru, and H12 of north-western and central Peru; Fig.2). Our results support the notion that pre-Pleistocene events underlie the initiation of genetic diversification in north-western Amazonia (Rull, 2008; Hoorn *et al*., 2010) because Amazonian lineages date from *c*. 12 Ma. The regional genetic differentiation found is likely to have been promoted by this long-residence time of *F. insipida* subsp. *insipida* in Amazonia, which means it was impacted upon by Andean uplift during the Miocene (*c*. 12 Ma) and early Pliocene (*c*. 4.5 Ma) (Hoorn *et al*., 1995, 2010) and the drying of Lake Pebas around 7 Ma (Hoorn *et al*., 2010).

### Demographic history in Amazonia

Despite the genetic differentiation of some *F. insipida* subsp. *insipida* populations in Amazonia, there are widespread haplotypes in both plastid and ITS markers. In particular, the lower variation found in ITS and the presence of a single widespread haplotype in Amazonia (Fig.3) are suggestive of effective nuclear gene flow via pollen. Pollen of *F. insipida* is dispersed by tiny aganoid wasps of the genus *Tetrapus* (Machado *et al*., 2001). Wasps that pollinate monoecious fig species such as *F. insipida* have been reported to travel over distances of up to 14 km in the Neotropics (Nason *et al*., 1998) and up to more than 150 km in riparian vegetation of the Namib Desert, Namibia (Ahmed *et al*., 2009), indicating that pollen dispersal may succeed even among geographically distant individuals. Effective gene flow via pollen has often been reported in pioneer tree species, indicating that diverse organisms including small vertebrates and insects are able to pollinate over long distances (Table4).

The population differentiation of *F. insipida* subsp. *insipida* shown by the plastid DNA data in north-western Amazonia suggests that seed flow must in some cases have been more restricted. Nevertheless, negative values of Fu's *F*_*S*_ and the results of mismatch distribution tests for both plastid and nuclear markers tentatively suggest that the SAMOVA group that contains most Amazonian populations (group VII) has experienced recent demographic expansion, most likely during the Pleistocene (Table3). Moreover, an interesting pattern of no genetic diversity is observed within the Bolivian populations for both markers. This uniformity, which is also present at the northernmost range in Mesoamerica for the plastid marker, probably reflects recent colonization events. This is consistent with palaeoecological data suggesting that the Amazon rain forest expanded south in the last 3000 years and that the current vegetation in the region (near population 54) may represent the southernmost distribution of rain forest over the last 50,000 years (Mayle *et al*., 2000). This southern margin of Amazonia has a marked dry season where monthly rainfall can be less than 100 mm for 4–6 months (Sombroek, 2001). If even drier conditions occurred during the Pleistocene, *F. insipida* subsp. *insipida* (and other rain forest trees) may have experienced range contraction at the southern end of its range and a reduction in effective population size. By contrast, the presence of populations containing high haplotype and nucleotide diversity (e.g. population 33 in Ecuador) for the plastid marker suggest that *F. insipida* subsp. *insipida* may have persisted in ‘refugia’ in wetter forest of north-western Amazonia.

The occurrence of widespread plastid haplotypes in some areas of the range of *F. insipida* subsp. *insipida* is consistent with episodes of effective seed dispersal. Fig fruits are important for frugivores in the Neotropics, and fish and bats are the primary seed dispersers of *F. insipida* (Banack *et al*., 2002). The mobility of these dispersal agents could help explain wide-scale dispersal. In particular, fish contribute significantly to the upstream dispersal of riparian plants (Reys *et al*., 2009), and fruit-eating bats are known to travel long distances from fruiting trees (Janzen, 1978; Pennington & de Lima, 1995). Successful establishment after dispersal is also favoured by the ecology of *F. insipida*, which is a light-demanding species that grows in riparian and disturbed areas (Berg, 2001; Banack *et al*., 2002).

## Conclusions

Our phylogeographical study of *F. insipida* subsp. *insipida* showed marked genetic differentiation between Amazonian and Mesoamerican populations in the plastid DNA marker *trn*H–*psb*A. This contrasts with previous studies of other Neotropical trees – for example *Cordia alliodora*, *Ceiba pentandra*, *Jacaranda copaia*, *Trema micrantha* – that have little differentiation between these areas. Although all these species are pioneers, only *F. insipida* subsp. *insipida* is confined to ever-wet habitats. This suggests that the ecological characteristics of species, in this case a requirement of moist conditions for regeneration and survival, seem to be important drivers of phylogeographical patterns in Neotropical trees. The tolerance of seasonally dry climates in the other species suggests that seasonally dry tropical forest and savannas in northern South America did not represent long-term barriers to their migration. In contrast, areas with seasonally dry climates may have significantly restricted seed dispersal of *F. insipida* subsp. *insipida*. Further phylogeographical studies of widespread species will be needed to determine the extent to which ecological characteristics have determined historical migration patterns of other Neotropical tree species.

Within Amazonia, the plastid marker of *F. insipida* subsp. *insipida* also shows genetic differentiation among populations of the northern basin. Pre-Pleistocene events related to the changes of the landscape caused by the uplift of the Andes may be responsible for the initiation of population differentiation there. In contrast, the tentative evidence for demographic expansion in the rest of the basin, in particular the presence of genetically uniform populations across southern Amazonia, may indicate more recent colonization events, a scenario consistent with the palaeoecological data that indicates post-glacial rain forest expansion close to the southern margins of the forest.

## Biosketch

**Eurídice Honorio** is a researcher at the Instituto de Investigaciones de la Amazonia Peruana, and has recently obtained her PhD at the University of Leeds, UK. Her research focuses on tropical ecology and she is interested in understanding the ecological and historical processes determining tree species distribution in Amazonia.

Author contributions: E.H., T.P. and O.P. conceived the idea; E.H., M.P. and K.D. collected the samples; E.H. and M.P. carried out the DNA lab work; E.H. performed statistical analyses; E.H. and T.P. wrote the paper; and K.D., M.P, P.H. and O.P. contributed to the writing. All authors read and approved the final manuscript.

## Supporting Information

**Appendix S1**List of sampled sites for fresh and herbarium material of *Ficus insipida* subsp. *insipida* **Appendix S2** (a) Phylogram and (b) haplotype network for all plastid DNA haplotypes (H1–H19) of *Ficus insipida* subsp. *insipida* occurring in Mesoamerica and Amazonia. **Appendix S3** Haplotypes and detected polymorphic sites for *trn*H*–psb*A and ITS of *Ficus insipida* subsp. *insipida*.
