# Supplementary material for: Ficus insipida subsp. insipida (Moraceae) reveals the role of ecology in the phylogeography of widespread Neotropical rain forest tree species
Source: J Biogeogr. 2014 May 14;41(9):1697–709. doi: 10.1111/jbi.12326 (PMC4368618; doi:10.1111/jbi.12326)
Supplement: Supplementary file 1 — Appendix S1List of sampled sites for fresh and herbarium material of Ficus insipida subsp. insipida Appendix S2 (a) Phylogram and (b) haplotype network for all plastid DNA haplotypes (H1–H19) of Ficus insipida subsp. insipida occurring in Mesoamerica and Amazonia. Appendix S3 Haplotypes and detected polymorphic sites for trnH–psbA and ITS of Ficus insipida subsp. insipida. [file jbi0041-1697-sd1.doc]

# Journal of Biogeography

# SUPPORTING INFORMATION

# Ficus insipida subsp. insipida (Moraceae) reveals the role of ecology in the phylogeography of widespread Neotropical rain forest tree species

Eurídice N. Honorio Coronado, Kyle G. Dexter, Monica F. Poelchau, Peter M. Hollingsworth, Oliver L. Phillips and R. Toby Pennington

**Appendix S1** List of sampled sites for fresh and herbarium material of *Ficus insipida* subsp. *insipida* in Mesoamerica and Amazonia. Localities of samples taken from herbarium specimens are highlighted with an asterisk. The last four sites indicate localities of additional ITS sequences obtained from collaborators (see acknowledgements). Collector codes: Euridice Honorio (EH), Kyle Dexter (KD) and Abel Monteagudo (AM). Mesoamerican samples (collected by M.F.P.) do not have voucher accession numbers.

| No. | Code | Region | Country | Population name | Lat | Long | Voucher (Herbarium code) | GenBank accession number (marker) |
| --- | --- | --- | --- | --- | --- | --- | --- | --- |
| 1 | MEX | Mesoamerica | Mexico | Rio Grijalva* | 16.56 | –92.78 | Accession # 1666497 (F) | KJ734283 (*trn*H–*psb*A) |
| 2 | BEL | Mesoamerica | Belize | Roaring Creek* | 12.23 | –88.80 | Accession # 1834951 (F) | KJ734284 (*trn*H–*psb*A) |
| 3 | ElI | Mesoamerica | El Salvador | El Imposible | 13.83 | –89.95 |  | GQ438036–438039, GQ438137–438140 (*trn*H–*psb*A) |
| 4 | Dei | Mesoamerica | El Salvador | Walter T. Deiniger | 13.48 | –89.27 |  | GQ438028–438031, GQ438129–438132 (*trn*H–*psb*A) |
| 5 | Nan | Mesoamerica | El Salvador | Nancuchiname | 13.34 | –88.71 |  | GQ438073–438076, GQ438173–438176 (*trn*H–*psb*A) |
| 6 | VoC | Mesoamerica | Nicaragua | Volcan Cosiguina | 13.00 | –87.63 |  | GQ438105–438111, GQ438205–438211 (*trn*H–*psb*A) |
| 7 | Mir | Mesoamerica | Nicaragua | Miraflor | 13.21 | –86.34 |  | GQ438069–438072, GQ438169–438172 (*trn*H–*psb*A) |
| 8 | ElO | Mesoamerica | Nicaragua | Zacatan | 11.44 | –85.94 |  | KJ734285–KJ734291 (*trn*H–*psb*A) |
| 9 | HLI | Mesoamerica | Costa Rica | Hac. Los Inocentes | 11.03 | –85.50 |  | GQ438051–438054, GQ438152–438155 (*trn*H–*psb*A) |
| 10 | RiT | Mesoamerica | Costa Rica | Rio Tempisquito | 10.79 | –85.55 |  | GQ438093–438096, GQ438192–438196 (*trn*H–*psb*A) |
| 11 | CaN | Mesoamerica | Costa Rica | Cano Negro | 10.89 | –85.00 |  | GQ438015–438018, GQ438116–438118 (*trn*H–*psb*A) |
| 12 | RiB | Mesoamerica | Costa Rica | Rio Bebedero | 10.34 | –85.21 |  | GQ438187 (*trn*H–*psb*A) |
| 13 | RiN | Mesoamerica | Costa Rica | Rio Nacaome | 10.18 | –85.37 |  | GQ438088–438092, GQ438188–438191 (*trn*H–*psb*A) |
| 14 | LaE | Mesoamerica | Costa Rica | La Ensenada | 10.14 | –85.04 |  | GQ438059, GQ438160 (*trn*H–*psb*A) |
| 15 | Cur | Mesoamerica | Costa Rica | Curu | 9.79 | –84.93 |  | GQ438024–438027, GQ438125–438128 (*trn*H–*psb*A) |
| 16 | CaB | Mesoamerica | Costa Rica | Cabo Blanco | 9.59 | –85.09 |  | KJ734292–KJ734294 (*trn*H–*psb*A) |
| 17 | RSC | Mesoamerica | Costa Rica | Rio San Carlos | 10.36 | –84.51 |  | GQ438097–438104, GQ438197–438204 (*trn*H–*psb*A) |
| 18 | Esp | Mesoamerica | Costa Rica | Esparza | 10.02 | –84.64 |  | GQ438040–438042, GQ438141–438143 (*trn*H–*psb*A) |
| 19 | Jac | Mesoamerica | Costa Rica | Jaco | 9.60 | –84.62 |  | GQ438055–438058, GQ438156–438159 (*trn*H–*psb*A) |
| 20 | LaS | Mesoamerica | Costa Rica | La Selva | 10.43 | –84.01 |  | GQ438060–438063, GQ438161–438164 (*trn*H–*psb*A) |
| 21 | EaU | Mesoamerica | Costa Rica | Earth University | 10.18 | –83.61 |  | GQ438032–438035, GQ438133–438136 (*trn*H–*psb*A) |
| 22 | Car | Mesoamerica | Costa Rica | Carara | 9.78 | –84.61 |  | GQ438019–438021, GQ438119–438121 (*trn*H–*psb*A) |
| 23 | MaA | Mesoamerica | Costa Rica | Manuel Antonio | 9.38 | –84.14 |  | GQ438068, GQ438168 (*trn*H–*psb*A) |
| 24 | HaB | Mesoamerica | Costa Rica | Hacienda Baru | 9.27 | –83.88 |  | GQ438047–438050, GQ438147–438151 (*trn*H–*psb*A) |
| 25 | Cah | Mesoamerica | Costa Rica | Cahuita | 9.70 | –82.88 |  | GQ438012–438014, GQ438112–438115 (*trn*H–*psb*A) |
| 26 | PiB | Mesoamerica | Costa Rica | Piedras Blancas | 8.70 | –83.21 |  | GQ438077–438079, GQ438177–438178 (*trn*H–*psb*A) |
| 27 | CeB | Mesoamerica | Panama | Cerro Batipa | 8.39 | –82.23 |  | GQ438022–438023, GQ438122–438124 (*trn*H–*psb*A) |
| 28 | LaT | Mesoamerica | Panama | La Tronosa | 7.37 | –80.46 |  | GQ438064–438067, GQ438165–438167 (*trn*H–*psb*A) |
| 29 | FtS | Mesoamerica | Panama | Ft. Sherman | 9.33 | –79.95 |  | GQ438043–438046, GQ438144–438146 (*trn*H–*psb*A) |
| 30 | PLR | Mesoamerica | Panama | Pipeline Road | 9.12 | –79.72 |  | GQ438080–438083, GQ438179–438183 (*trn*H–*psb*A) |
| 31 | PNM | Mesoamerica | Panama | Metropolitana | 8.99 | –79.54 |  | GQ438084–438087, GQ438184–438186 (*trn*H–*psb*A) |
| 32 | JaS | Amazonia | Ecuador | Jatun Sacha | –1.07 | –77.62 | EH654, 702, 710–717 (HOXA) | KJ734295–KJ734304 (*trn*H–*psb*A), KJ734485–KJ734490 (ITS) |
| 33 | Bog | Amazonia | Ecuador | Bogi | –0.70 | –76.48 | AM19374, AM19649, 19650, 19563 (HOXA) | KJ734305–KJ734308 (*trn*H–*psb*A), KJ734491–KJ734492 (ITS) |
| 34 | Yan | Amazonia | Peru | Yanamono | –3.44 | –72.85 | EH977–986 (MOL), Sandra Patiño s.n. | KJ734309–KJ734319 (*trn*H–*psb*A), KJ734493–KJ734497 (ITS) |
| 35 | Mad | Amazonia | Peru | Madreselva | –3.62 | –72.25 | EH988–992, 1025, 1042, 1054–1056, KD9 (MOL) | KJ734320–KJ734330 (*trn*H–*psb*A), KJ734498–KJ734502 (ITS) |
| 36 | SaJ | Amazonia | Peru | San Jorge | –4.06 | –73.20 | EH913–918 (MOL) | KJ734331–KJ734336 (*trn*H–*psb*A), KJ734503–KJ734507 (ITS) |
| 37 | JeH | Amazonia | Peru | Jenaro Herrera | –4.90 | –73.65 | EH948, 954–960, 975 (MOL) | KJ734337–KJ734345 (*trn*H–*psb*A), KJ734508–KJ734512 (ITS) |
| 38 | Mar | Amazonia | Peru | Maray | –6.31 | –76.66 | EH1164,1169, 1170, 1172–1178 (MOL) | KJ734346–KJ734355 (*trn*H–*psb*A), KJ734513–KJ734516 (ITS) |
| 39 | Ura | Amazonia | Peru | Urahuacha | –6.47 | –76.33 | EH1127, 1132, 1135, 1138, 1139, 1142–1144, 1149, 1151 (MOL) | KJ734356–KJ734365 (*trn*H–*psb*A), KJ734517–KJ734521 (ITS) |
| 40 | vHu | Amazonia | Peru | von Humboldt | –8.83 | –75.06 | EH1219–1221, 1223–1226, 1233, 1235, 1236 (MOL) | KJ734366–KJ734375 (*trn*H–*psb*A), KJ734522–KJ734526 (ITS) |
| 41 | Mac | Amazonia | Peru | Macuya | –8.87 | –75.01 | EH1238–1241, 1248–1251, 1254, 1256 (MOL) | KJ734376–KJ734385 (*trn*H–*psb*A), KJ734527–KJ734530 (ITS) |
| 42 | LaG | Amazonia | Peru | La Genova | –11.09 | –75.35 | EH505–508, 547–552 (MOL) | KJ734386–KJ734395 (*trn*H–*psb*A), KJ734531–KJ734533 (ITS) |
| 43 | SaT | Amazonia | Peru | Santa Teresa | –11.17 | –74.66 | EH553, 556–565 (MOL) | KJ734396–KJ734406 (*trn*H–*psb*A), KJ734534–KJ734538 (ITS) |
| 44 | CoC | Amazonia | Peru | Cocha Cashu | –11.90 | –71.36 | KD167–170 (MOL) | KJ734407–KJ734410 (*trn*H–*psb*A), KJ734539–KJ734542 (ITS) |
| 45 | Qon | Amazonia | Peru | Qoñec | –12.90 | –71.37 | EH1294,1318–1325, 1335, 1341 (MOL) | KJ734411–KJ734421 (*trn*H–*psb*A), KJ734543–KJ734546 (ITS) |
| 46 | SaG | Amazonia | Peru | San Gaban | –13.50 | –70.42 | Miguel Luza 611 (photographic material only) | KJ734422 (*trn*H–*psb*A), KJ734547 (ITS) |
| 47 | Tam | Amazonia | Peru | Tambopata | –11.16 | –68.71 | EH1641–1645, KD3554–3556 (MOL) | KJ734423–KJ734429 (*trn*H–*psb*A), KJ734548–KJ734549 (ITS) |
| 48 | LoA | Amazonia | Peru | Los Amigos | –12.57 | –70.10 | KD45, 224, 259, 260 (MOL) | KJ734430–KJ734433 (*trn*H–*psb*A), KJ734550–KJ734553 (ITS) |
| 49 | LaP | Amazonia | Peru | Las Piedras | –12.06 | –69.53 | KD3974 (MOL) | KJ734434 (*trn*H–*psb*A), KJ734554 (ITS) |
| 50 | Tah | Amazonia | Bolivia | Tahuamanu | –10.59 | –68.98 | EH1382, 1406–1410 (Herbario de Referencia Amazónica) | KJ734435–KJ734440 (*trn*H–*psb*A), KJ734555 (ITS) |
| 51 | Aba | Amazonia | Bolivia | Abaroa | –10.85 | –66.53 | EH1415, 1439, 1443–1446 (Herbario de Referencia Amazónica) | KJ734441–KJ734446 (*trn*H–*psb*A), KJ734556 (ITS) |
| 52 | Mai | Amazonia | Bolivia | Maije | –13.65 | –66.32 | EH1494–1503 (USZ) | KJ734447–KJ734456 (*trn*H–*psb*A), KJ734557 (ITS) |
| 53 | Sac | Amazonia | Bolivia | Sacta | –16.90 | –63.22 | EH1559, 1560, 1562–1568, 1570 (USZ) | KJ734457–KJ734466 (*trn*H–*psb*A), KJ734558–KJ734559 (ITS) |
| 54 | LaEn | Amazonia | Bolivia | La Envidia | –16.32 | –62.41 | EH1573, 1574, 1611,1612,1617–1622 (USZ) | KJ734467–KJ734476 (*trn*H–*psb*A), KJ734560–KJ734561 (ITS) |
| 55 | MX | Mesoamerica | Mexico | Mexico | 16.56 | –92.78 | Otilene dos Anjos Santos 212–Fsp319 (INPA) | KJ734477 (ITS) |
| 56 | CR | Mesoamerica | Costa Rica | Costa Rica* | 10.59 | –84.02 | J–Y. Rasplus s.n.–Fsp293 (INPA) | KJ734478 (ITS) |
| 57 | BCI | Mesoamerica | Panama | Panama | 9.16 | –79.85 | Chris Dick s.n.–bci609355, bci636150, bci646282, bci721338, bci749198 (STRI) | KJ734479–KJ734483 (ITS) |
| 58 | BR | Amazonia | Brazil | Brazil | –3.18 | –60.18 | Otilene dos Anjos Santos 177–Fsp414 (INPA) | KJ734484 (ITS) |

**Appendix S2** (a) Phylogram and (b) haplotype network for all plastid DNA haplotypes (H1–H19) of *Ficus insipida* subsp. *insipida* occurring in Mesoamerica and Amazonia. Inversions and indels were excluded from both analyses. Branch lengths in the phylogram are given in units of substitutions per site. Sequences downloaded from GenBank are indicated in grey (including an additional sequence of *Ficus insipida* subsp. *insipida* collected in Barro Colorado Island, Panama).

(a)


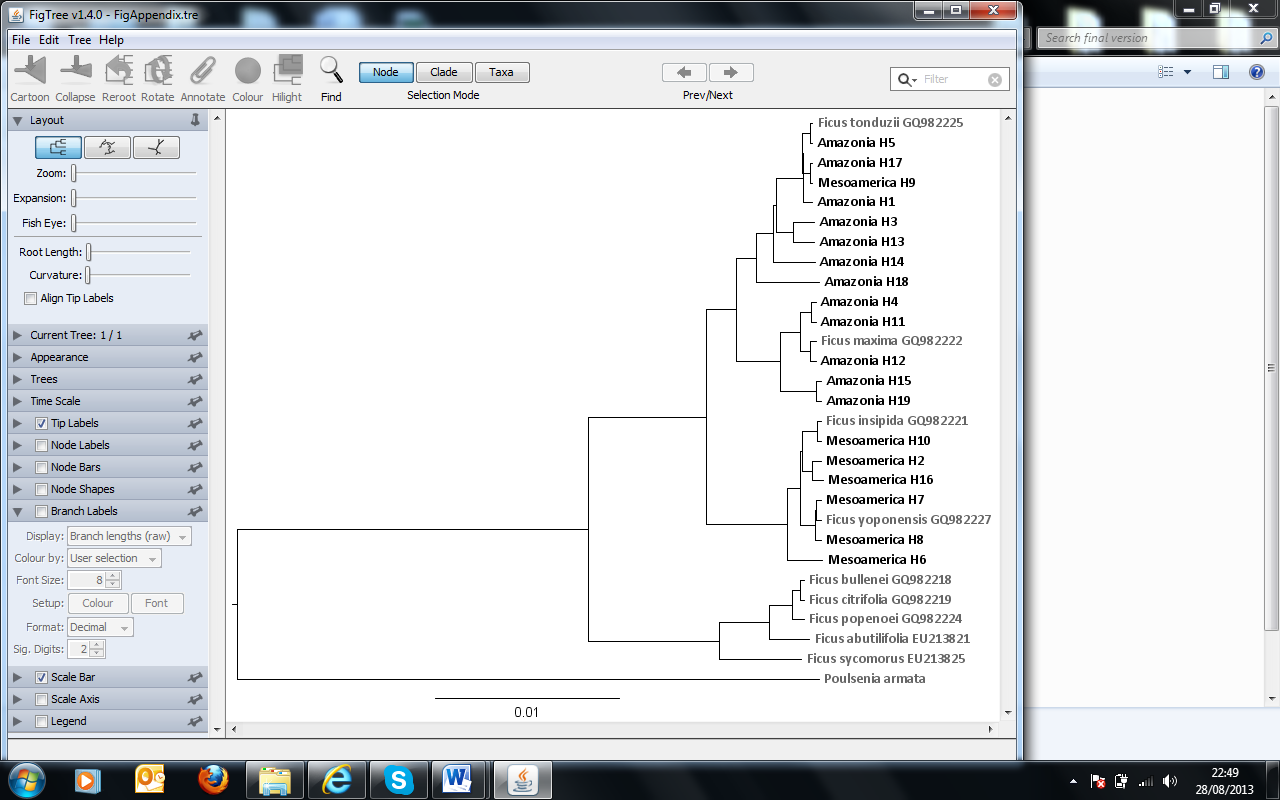


(b)

**Amaz H3**

***F. yoponensis***

***F. insipida***

**Meso H2**

**Meso H7**

**Meso H8**

**Meso H10**

**Meso H6**

**Meso H16**

**Amaz H13**

***F. tonduzii***

**Meso H9**

**Amaz H1**

**Amaz H5**

**Amaz H17**

**Amaz H15**

**Amaz H19**

***F. maxima***

**Amaz H4**

**Amaz H11**

**Amaz H12**

**Amaz H14**

**Amaz H18**

***F. sycomorus***

***F. bullenei***

***F. citrifolia***

***F. popenoei***

***F. abutilifolia***

**Appendix S3** Haplotypes and detected polymorphic sites for *trn*H*–psb*A and ITS found on populations of *Ficus insipida* subsp. *insipida* of Mesoamerica and Amazonia. ID = insertion/deletion, NS = nucleotide substitution, IV = inversion.

| Hapl. | *trn*H*–psb*A (375 base pairs) | | | | | | | | | | | | | | | | | | | ITS (635 bp) | | | | |
| --- | --- | --- | --- | --- | --- | --- | --- | --- | --- | --- | --- | --- | --- | --- | --- | --- | --- | --- | --- | --- | --- | --- | --- | --- |
|  | ID  045 | NS  050 | I  V  060 | NS  075 | ID  076 | NS  087 | ID  100 | NS  111 | NS  120 | NS  122 | ID  135 | NS  165 | NS  245 | NS  248 | NS  258 | NS  267 | NS  300 | NS  325 | NS  344 | NS224 | ID406 | ID407 | NS512 | NS580 |
| H1 | - | A | T | T | - | G | - | T | A | T | - | T | A | T | T | A | T | G | T | T | - | - | T | T |
| H2 | - | A | T | T | - | G | - | T | A | T | - | C | A | T | T | A | T | G | C | G | - | - | T | T |
| H3 | - | A | T | T | - | G | - | T | A | T | - | T | A | T | T | A | T | T | T | T | C | C | T | T |
| H4 | A | A | T | T | A | G | - | T | A | T | - | T | A | T | T | G | T | G | T | T | C | C | T | C |
| H5 | - | A | A | T | - | G | - | T | A | T | - | T | A | T | T | A | T | G | T | T | - | - | C | T |
| H6 | - | A | T | T | - | G | - | T | A | A | - | C | A | T | T | A | T | G | C | T | C | - | T | T |
| H7 | - | A | A | T | - | G | C | T | A | T | - | C | A | T | T | A | T | G | C |  |  |  |  |  |
| H8 | - | A | A | T | - | G | - | T | A | T | - | C | A | T | T | A | T | G | C |  |  |  |  |  |
| H9 | - | A | T | T | - | G | - | T | A | T | - | T | C | A | T | A | T | G | T |  |  |  |  |  |
| H10 | - | A | T | T | - | G | C | T | A | T | - | C | A | T | T | A | T | G | C |  |  |  |  |  |
| H11 | A | A | A | T | A | G | - | T | A | T | - | T | A | T | T | G | T | G | T |  |  |  |  |  |
| H12 | - | A | T | T | A | G | - | T | A | T | - | T | A | T | T | G | T | G | T |  |  |  |  |  |
| H13 | - | A | T | T | - | G | - | T | T | T | - | T | A | T | T | A | T | G | T |  |  |  |  |  |
| H14 | - | A | A | T | - | G | - | A | A | T | - | T | A | T | T | A | T | G | T |  |  |  |  |  |
| H15 | A | C | A | G | A | G | - | T | A | T | - | T | A | T | T | G | T | G | T |  |  |  |  |  |
| H16 | - | A | T | T | - | G | - | T | A | T | - | C | A | T | T | A | G | G | C |  |  |  |  |  |
| H17 | - | A | T | T | - | G | - | T | A | T | T | T | A | T | T | A | T | G | T |  |  |  |  |  |
| H18 | - | A | T | T | - | A | - | T | A | T | - | T | A | T | A | A | T | G | T |  |  |  |  |  |
| H19 | A | C | T | G | A | G | - | T | A | T | - | T | A | T | T | G | T | G | T |  |  |  |  |  |

Coded sites for *trn*H*–psb*A: ID045: -/ AAAAT, IV060: TTCTAT/ ATAGAA, ID076: -/ ATTTT, ID100: -/ CATTTT, ID135: -/ TATTTGTCTTTT. Excluded sites for *trn*H*–psb*A: ID171: poly-T, ID253: poly-A.
